# Distinct affective neural phenotypes in breast cancer survivors: implications for personalized supportive care after chemotherapy

**DOI:** 10.1007/s00520-026-11271-z

**Published:** 2026-10-02

**Authors:** Chenyi Chen, Hao-Jin Chiang, Yu-Chun Chen, Yang-Teng Fan, Tsai-Tsen Liao, Chin-Yau Chen, Yawei Cheng

**Affiliations:** 1 Graduate Institute of Injury Prevention and Control, College of Public Health, Taipei Medical University, Taipei, Taiwan; 2 Psychiatric Research Center, Wan Fang Hospital, Taipei Medical University, Taipei, Taiwan; 3 Graduate Institute of Mind, Brain and Consciousness, College of Humanities and Social Sciences, Taipei Medical University, Taipei, Taiwan; 4 Research Center for Mind, Brain & Learning, National Chengchi University, Taipei, Taiwan; 5 Institute of Neuroscience and Brain Research Center, National Yang Ming Chiao Tung University, No. 155, Sec. 2, Linong St. Beitou District, Taipei, 112 Taiwan, ROC; 6 Department of Physical Education, National Taiwan University of Sport, Taichung, Taiwan; 7 Graduate Institute of Medicine, Yuan Ze University, Taoyuan City, Taiwan; 8 Graduate Institute of Medical Sciences, College of Medicine, Taipei Medical University, Taipei, Taiwan; 9 Research Center of Cancer Translational Medicine, Taipei Medical University, Taipei, Taiwan; 10 Department of Surgery, National Yang Ming Chiao Tung University Hospital, Siaoshe Rd, No. 169, Yilan, 26058 Taiwan, ROC; 11 Department of Physical Medicine & Rehabilitation, National Yang Ming Chiao Tung University Hospital, Yilan, Taiwan; 12 Department of Education and Research, Taipei City Hospital, Taipei, Taiwan; 13 Cell Physiology and Molecular Image Research Center, Wan Fang Hospital, Taipei Medical University, Taipei, Taiwan; 14 Center for Precision Medicine and Translational Cancer Research, Taipei Medical University Hospital, Taipei, Taiwan; 15 Research Center of Sleep Medicine, Taipei Medical University Hospital, Taipei, Taiwan; 16 Master Program in Graduate Institute of Clinical Psychology, College of Medicine, Taipei Medical University, Taipei, Taiwan

**Keywords:** Breast cancer survivorship, Supportive care, Chemotherapy, Emotional blunting, Distress screening, Functional MRI

## Abstract

**Purpose:**

Anxiety and emotional dysregulation remain among the most prevalent unmet supportive care needs in breast cancer survivorship, yet reported affective outcomes are strikingly inconsistent, with some survivors showing elevated distress and others displaying resilience or post-traumatic growth. Because distress screening in survivorship relies almost exclusively on self-report, the neural mechanisms shaping how chemotherapy-exposed survivors perceive and report emotional experience remain poorly understood, limiting mechanism-informed psychosocial support.

**Methods:**

Forty female breast cancer survivors (27 chemotherapy-treated, 13 non-chemotherapy) and 30 age-matched healthy controls completed a validated face-word emotional Stroop task during functional MRI to probe emotional attention and conflict regulation. Standardized cognitive and affective questionnaires were aggregated into composite indices characterizing each survivor’s neurobehavioral profile.

**Results:**

Chemotherapy-treated survivors showed poorer Stroop accuracy for faces normatively categorized as fearful and hypoactivation of the dorsomedial prefrontal cortex, yet reported lower affective symptoms than non-chemotherapy survivors, who instead showed heightened negative attentional bias and greater incongruency-related interference in the parahippocampal gyrus and insula. A normative inverse coupling between dorsal anterior cingulate cortex activation and affective symptom severity was present only in healthy controls.

**Conclusion:**

Breast cancer survivors exhibit distinct neurobehavioral phenotypes with potential supportive care implications. Lower self-reported distress in chemotherapy-treated survivors paralleled reduced salience network engagement, a configuration compatible with emotional blunting rather than genuine well-being; because emotional blunting was not measured directly, this remains an interpretive hypothesis. These findings caution against relying solely on self-report screening and motivate the hypothesis that treatment-history-informed survivorship care may be beneficial—affective re-engagement strategies for chemotherapy-exposed survivors, and attention-bias modification or mindfulness-based approaches for non-chemotherapy survivors—a proposal that now requires testing in intervention trials.

**Supplementary Information:**

The online version contains supplementary material available at https://doi.org/10.1007/s00520-026-11271-z.

## Introduction

Breast cancer survivorship is increasingly defined not only by disease-free survival but by the quality of life that follows treatment. Anxiety, depression, and emotional dysregulation remain among the most prevalent unmet supportive care needs in this population, affecting daily functioning, follow-up adherence, and long-term psychosocial recovery. Despite extensive attention to the cognitive sequelae of chemotherapy—commonly termed “chemo-fog” or “chemo brain”—the affective consequences of treatment remain comparatively underexplored [23]. Chemotherapy-related impairments in memory, attention, executive function, and processing speed are well established and have been linked to structural and functional alterations in prefrontal cortex and hippocampus [9, 24, 26, 35]. Yet the emotional consequences of chemotherapy—particularly emotional attention, mood regulation, and the affective neural circuitry that supports them—have received far less systematic investigation, despite being central to the symptoms that oncology supportive care teams are most often asked to address. Current distress screening in survivorship relies almost exclusively on self-report, yet the neural substrates shaping how survivors perceive and report emotional experience remain largely unknown, limiting mechanism-informed, personalized supportive care. To our knowledge, no prior study has employed task-based fMRI with an affective paradigm specifically designed to probe emotional attention and conflict processing in breast cancer survivors.

Compounding this gap, the supportive care literature reports strikingly divergent affective trajectories. On one hand, many survivors show enhanced resilience, improved mood regulation, and post-traumatic growth (PTG) [17, 18, 25, 34]. On the other, elevated anxiety, depression, fatigue, and psychological distress are widely documented, particularly after chemotherapy, with a small number of neuroimaging studies revealing disruptions in parieto-frontal networks implicated in affective regulation that occur independently of cognitive load [2, 6, 20]. For supportive care providers, these contradictory outcomes create a clinical dilemma: self-reported affective scores alone may not reliably identify survivors at greatest neurobiological risk, nor distinguish genuine psychological adaptation from attenuated emotional reactivity.

Emerging electrophysiological evidence offers clues. In breast cancer survivors, emotion detection tasks show prolonged N1 and P2 latencies, indicating compromised early-stage attentional engagement with emotional stimuli [14]. Because heightened emotional attention can itself amplify emotional burden [17], reduced emotional attention after chemotherapy may paradoxically function as an inadvertent buffer—lowering the salience of emotional stressors and appearing on self-report as resilience. Whether this reflects genuine well-being or masked vulnerability has direct consequences for how supportive care teams interpret low distress scores in chemotherapy-exposed survivors.

We therefore propose a mechanistic framework centered on emotional attention disruption, quantifiable using the emotional Stroop task during conflict monitoring and affective processing [7]. We hypothesized that chemotherapy impairs neural circuits encoding emotional salience and attentional bias—including the insula, parahippocampal region, anterior cingulate cortex (ACC), and dorsomedial prefrontal cortex (DMPFC) [13, 16, 31]—manifesting as emotional blunting: a dampening of neural and behavioral sensitivity to emotionally salient stimuli that may be misinterpreted as resilience. To test this, we compared chemotherapy-treated survivors (CHT), non-chemotherapy survivors (BC), and healthy controls on an emotional Stroop task during fMRI. We predicted that, alongside cognitive impairments, CHT survivors would show lower affective symptomatology accompanied by blunted Stroop interference, reduced prefrontal monitoring activity, or compensatory activity in salience regions. By characterizing the neurobehavioral phenotypes underlying divergent affective outcomes, we aim to provide the mechanistic evidence needed to move beyond one-size-fits-all distress screening toward treatment history-informed, personalized psychosocial support.

## Terminology

Because several closely related constructs recur throughout this report, we use them in the following restricted senses. Emotional attention denotes the preferential allocation of attentional resources to emotionally salient stimuli. Emotional conflict denotes competition simultaneously presented, incongruent emotional emotional cues (here, face and word), indexed by the congruency (incongruent–congruent) contrast. Negative attentional bias denotes the processing cost or advantage associated with negatively relative to positively valenced stimuli, indexed by the emotion (fearful–happy) contrast. Salience processing denotes the detection and prioritization of behaviorally relevant stimuli, supported by insula- and cingulate-centered circuitry. Emotional blunting denotes an attenuation of affective reactivity across neural, behavioral, and subjective levels; in the present study, it is treated as an interpretive hypothesis rather than as a directly measured variable. Finally, faces described as “happy” or “fearful” denote standardized stimuli that are normatively categorized as such, not verified internal emotional states of the depicted individuals.

## Materials and methods

### Participants

Forty female breast cancer survivors (27 chemotherapy-treated) and 30 healthy controls were enrolled. Survivors were recruited through National Yang Ming Chiao Tung University Hospital, with diagnoses clinically confirmed by physicians. Chemotherapy regimens consisted primarily of AC (doxorubicin and cyclophosphamide) or FEC (5-fluorouracil, epirubicin, and cyclophosphamide), with some patients receiving additional agents such as docetaxel. Healthy controls were recruited from the community and screened via recent health examination records. All participants were right-handed, literate Taiwanese women aged 35–75 with normal or corrected-to-normal vision. Individuals with a history of neurological or psychiatric disorders, substance abuse, metal implants, or claustrophobia were excluded. Written informed consent was obtained in accordance with the Declaration of Helsinki; the protocol was approved by the IRB of National Yang Ming Chiao Tung University Hospital (No. 2021B002).

### Clinical neuropsychological assessment

Participants completed the Chinese Montreal Cognitive Assessment (MoCA) [5] and Digit Span Test (DST) (Yang et al. 2012 [37] for cognition and the Chinese Beck Anxiety Inventory (BAI) [21] and Beck Depression Inventory-II (BDI-II) [33] for emotional distress (Cronbach’s *α* = 0.775–0.935). A cognitive index was derived by z-transforming and aggregating MoCA and DST scores; BAI and BDI-II scores were summed to form an affective index.

#### Face-word emotional Stroop task

Participants performed an emotional face-word Stroop task consisting of eight “on” and eight “off” blocks [7]. “On” blocks alternated between four congruent (CON) and four incongruent (INCON) blocks (14 trials each; mean duration 15.44 ± 4.4 s). Each trial presented a happy or fearful emotional word (bold red font) superimposed on one of 20 facial expressions from the Pictures of Facial Affect [10]. In CON trials, the word and face conveyed the same emotion; in INCON trials, they were mismatched. Each trial began with a 200-ms fixation cross, followed by a 400-ms blank screen and a 4-s stimulus display. Participants identified the facial expression (happy vs. fearful) while ignoring the word. A 10-trial practice session preceded scanning. Stimuli were presented using MATLAB (MathWorks Inc., Sherborn, MA). Full MRI acquisition parameters and preprocessing are detailed in the Supplementary Materials. The stimulus set comprised 20 photographs from the Pictures of Facial Affect: 10 identities displaying a prototypically happy expression and 10 identities displaying a prototypically fearful expression, selected on the basis of the normative recognition rates reported for the validated set and previously used with this paradigm [7]. The identical stimulus set was presented to every participant. Trial order within each block, and the pairing of happy or fearful words with individual face photographs, was pseudorandomized for each participant under the constraint that every face appeared equally often in congruent and incongruent trials across the task; response-hand mapping was counterbalanced across participants. Throughout this report, “happy” and “fearful” faces are used as shorthand for standardized facial stimuli normatively categorized as happy or fearful and not as claims about the internal emotional states of the depicted individuals.

#### Data analysis

Behavioral analyses were performed in SPSS v24.0. Trials with errors or reaction times (RTs) outside 300–4000 ms (< 0.5% of data) were excluded. A mixed-design ANOVA tested group (CHT, BC, Control), congruency (CON/INCON), and emotion (happy/fearful) effects on accuracy and RT. Because group sizes were unequal, homogeneity of variance was examined with Levene’s test and sphericity with Mauchly’s test, with Greenhouse-Geisser correction applied where sphericity was violated. For clinical measures whose variances differed markedly across groups (most notably the BAI), Welch’s ANOVA and Kruskal-Wallis tests were run as distribution-free sensitivity analyses.

Functional MRI data were analyzed using a two-level GLM in SPM12 (MATLAB R2023a). First-level voxel-wise models included CON and INCON task regressors convolved with the canonical HRF, with six motion parameters as nuisance regressors and a 128-s high-pass filter. Head motion was screened before second-level analysis: framewise displacement was computed from the realignment parameters, and data were required to show mean framewise displacement < 0.5 mm and maximum translation/rotation < 3 mm/3°. No participant exceeded these criteria, so no participant was excluded for motion, and volume censoring (scrubbing) was not applied; the six realignment parameters were retained as nuisance covariates. Second-level analyses paralleled the behavioral factorial design, with group as a between-subjects factor and congruency and emotion as within-subject factors, and with covariate analyses assessing brain-index associations. Because the groups differed in age, age was entered as a nuisance covariate in every second-level model, for both whole-brain and ROI analyses. Main effects and interactions were each thresholded and corrected within their own contrast rather than jointly across the family of contrasts, and results are interpreted accordingly.

Whole-brain results were thresholded at voxel-level *p* < 0.001 (uncorrected) with cluster-extent FWE correction (*p* < 0.05) determined by Monte Carlo simulation in 3dClustSim. Given the smoothness of the present data (8-mm FWHM Gaussian kernel), the voxel size, first-nearest-neighbor clustering, and the whole-brain search volume, the simulation returned a minimum cluster extent of *k* = 10 voxels for a cluster-level FWE-corrected *α* < 0.05. Every cluster reported in Table 2 therefore meets the stated corrected threshold; because this corrected extent threshold is small, clusters near the limit are interpreted with corresponding caution. Anatomical labels for whole-brain clusters were assigned with the Talairach Daemon after MNI-to-Talairach transformation.

A priori ROIs were defined as 10-mm-radius spheres centered on peak coordinates from previous meta-analyses [13, 16, 31]: DMPFC (6, 62, 14), dACC (−6, 14, 26), parahippocampal cortex (−12, −44, −2), and left insula (−34, −30, 22), analyzed with MarsBaR. A 10-mm radius was adopted because it approximates the spatial uncertainty of meta-analytic coordinates and is commensurate with the 8-mm smoothing kernel applied to these data, so that smaller spheres would sample fewer effectively independent voxels than the data resolution supports. The four spheres did not overlap: the smallest center-to-center separation, between the parahippocampal and insular ROIs, was 35.4 mm, well beyond the 20 mm required for two 10-mm spheres to be disjoint. All ROI coordinates reported in the text, figures, and Supplementary Materials refer to these a priori sphere centers; whole-brain peaks in Table 2 are reported separately and may differ from them. Because four ROIs were interrogated, ROI-level omnibus tests were additionally evaluated after Benjamini-Hochberg false-discovery-rate (FDR) correction across the seven reported ROI effects, and both uncorrected *p* values and FDR-corrected *q* values are given. Full preprocessing, first-level GLM specification, and ROI/statistical thresholding details are provided in the Supplementary Materials. Statistical significance was *p* < 0.05 throughout.

## Results

### Demographic and clinical characteristics

**Fig. 1:**
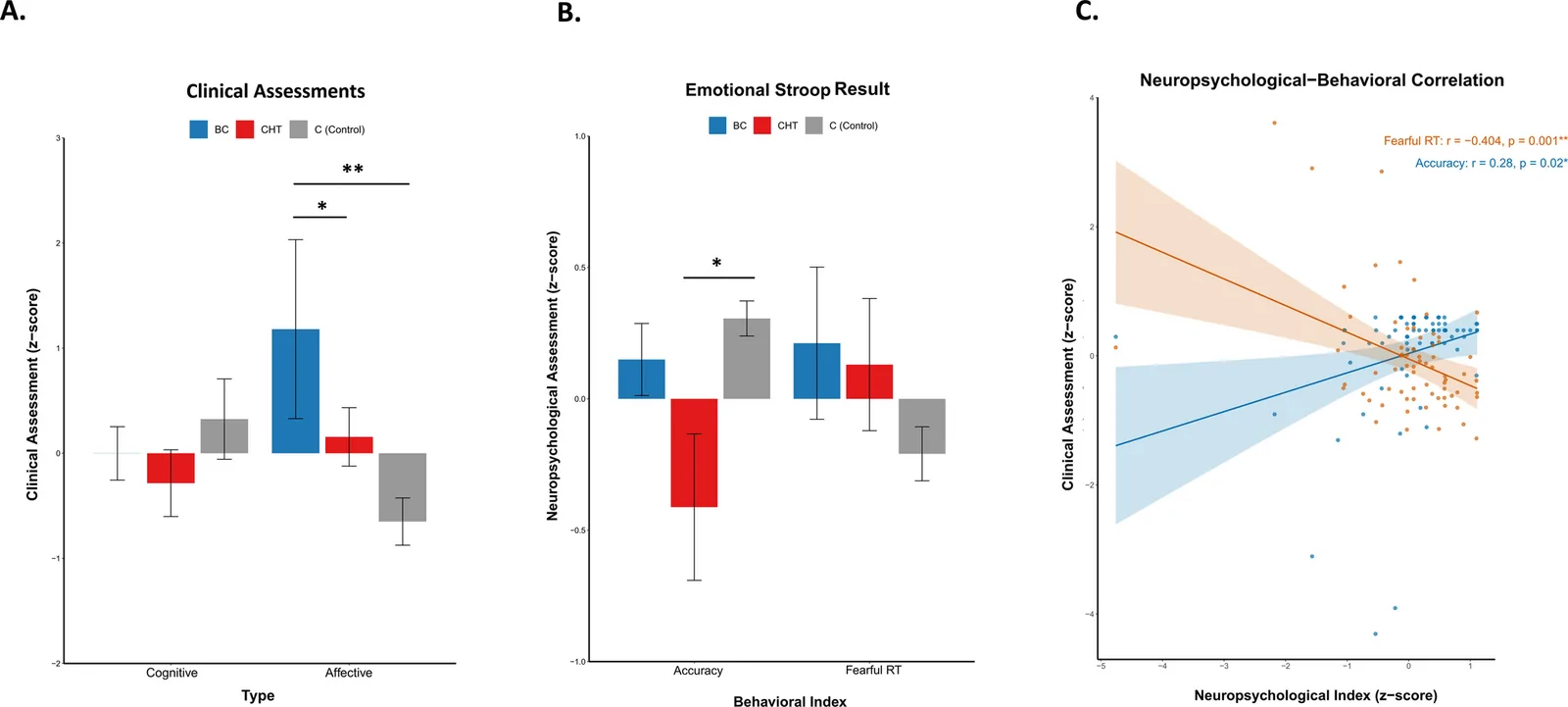
Neuropsychological outcomes and task performance across groups. **A** Neuropsychological indices, including cognitive and affective scores. **B** Task performance measures: accuracy and reaction time (RT) for trials with faces normatively categorized as fearful. **C** Correlations between neuropsychological indices and task performance. Groups are displayed from left to right as follows: BC (blue), CHT (red), and C (grey). All values are presented as mean ± standard error (SE). Asterisks denote statistically significant differences: *p* < 0.05 (*), *p* < 0.01 (**)

Demographics are summarized in Table 1. All values in Table 1 are raw, unadjusted means ± SD; one participant in the CHT group did not complete the DST, so cognitive index data were available for 69 participants. The three groups were comparable in menopausal status but differed in age. A two-way ANCOVA (group × index type, with age as covariate) revealed a significant main effect of group, *F*(2,65) = 3.26, *p* = 0.045, ηp2 = 0.091, and a group × index-type interaction, *F*(2,65) = 3.59, *p* = 0.033, ηp2 = 0.099. Post hoc, groups did not differ on the cognitive index (*p* = 0.554), but a one-way ANCOVA on the affective index was significant, *F*(2,66) = 6.144, *p* = 0.004, ηp2 = 0.157: the BC group showed the highest affective symptom load (raw affective index 1.18 ± 3.07) compared with both CHT (0.16 ± 1.45) and controls (–0.65 ± 1.23), indicating greater self-reported distress in non-chemotherapy survivors (Fig. 1A; Supplementary Table S1). The same ordering was present in both component scales (BDI-II: BC 9.46 ± 8.92, CHT 6.44 ± 4.47, C 5.20 ± 4.81; BAI: BC 13.23 ± 13.60, CHT 8.93 ± 7.21, C 3.87 ± 4.52).

**Table 1:** Demographic and neuropsychological characteristics of participants. Neuropsychological measures are presented for each group, along with ANCOVA results (*p*-values)

	BC (*N* = 13)	CHT (*N* = 27)	C (*N* = 30)	*p* value
Age (yr)	60.54 ± 10.76	55.22 ± 9.82	50.47 ± 10.42	0.013 *
Postmenopausal (%) ^	6 (46.15)	18 (67)	17 (56.67)	0.449
Cognitive Index^a^	0 ± 0.92	− 0.29 ± 1.65	0.32 ± 2.09	0.437
Affective Index	1.18 ± 3.07	0.16 ± 1.45	− 0.65 ± 1.23	0.004**
MoCA	25.69 ± 2.56	24.81 ± 4.33	27.27 ± 5.68	0.151
DST^a^	21.54 ± 3.45	20.65 ± 5.09	21.53 ± 5.41	0.78
* DSF*^a^	13.85 ± 1.52	13.07 ± 2.30	13.37 ± 3.31	0.7
*DSB*^a^	7.69 ± 2.72	7.62 ± 3.257	8.17 ± 2.93	0.772
BDI-II	9.46 ± 8.92	6.44 ± 4.47	5.20 ± 4.81	0.084
BAI	13.23 ± 13.6	8.93 ± 7.21	3.87 ± 4.52	0.002**

Asterisks denote statistical significance: *p* < 0.05 (*), *p* < 0.01 (**). All tabulated values are raw, unadjusted means ± SD; age-adjusted estimated marginal means are not tabulated, and the accompanying *p* values derive from ANCOVA with age as a covariate. (a) One participant in the CHT group did not complete the DST, so cognitive index, DST, DSF, and DSB data were available for 69 participants

*MoCA* Montreal Cognitive Assessment, *DST* Digit Span Test, comprising *DSF* (Digit Span Forward) and *DSB* (Digit Span Backward), with DST as the combined score of DSF and DSB; *BDI-II* Beck Depression Inventory-II (1996 revision), *BAI* Beck Anxiety Inventory. Values are reported as mean ± standard deviation (SD)

### Emotional Stroop task performance

Accuracy showed main effects of congruency (*F*(1,67) = 8.18, *p* = 0.006, ηp2 = 0.11; CON > INCON) and emotion (*F*(1,67) = 4.33, *p* = 0.041, ηp2 = 0.06; happy > fearful), plus a main effect of group (*F*(2,67) = 4.09, *p* = 0.021, ηp2 = 0.11). CHT performed worse than controls overall (0.898 ± 0.019 vs. 0.969 ± 0.018, *p* < 0.05; Fig. 1B), an effect driven by fearful faces (0.872 ± 0.037 vs. 0.958 ± 0.009, *p* < 0.05); happy face accuracy did not differ across groups. RT showed main effects of congruency (*F*(1,67) = 77.71, *p* < 0.001, ηp2 = 0.54) and emotion (*F*(1,67) = 37.57, *p* < 0.001, ηp2 = 0.36) but no overall group effect (*p* > 0.3). For fearful faces, a graded pattern emerged (BC 918 ms > CHT 897 ms > Controls 810 ms; Fig. 1B). Higher accuracy (*r* = 0.28, *p* = 0.02) and shorter fearful-face RTs (*r* = –0.404, *p* = 0.001) correlated with better cognitive index scores (Fig. 1C).

### fMRI results

Whole-brain activations are summarized in Table 2. All clusters survived a voxel-level threshold of *p* < 0.001 together with the Monte Carlo–derived minimum cluster extent of *k* ≥ 10 voxels, corresponding to a cluster-level FWE-corrected *p* < 0.05; each main effect was corrected separately. Emotional conflict (INCON > CON) engaged the ACC with decreases in temporal/parahippocampal regions; negative attentional bias (fearful > happy) engaged parahippocampal, precentral, and middle frontal regions.

**Table 2:** Main effects of congruency and emotion on neural activation

Brain region	*x*	*y*	*z*	Peak *T*	cluster size
**(A) Increased activation during interference effect (*incongruent* > *congruent*)**					
Left anterior cingulate gyrus (ACC, BA32)	− 16	− 26	28	3.55	12
Right corpus callosum	16	− 32	22	3.53	31
**(B) Decreased activation during interference effect (*incongruent* < *congruent*)**					
Right middle temporal gyrus (BA21)	56	− 26	− 6	5.15	3043
Left precuneus (BA7)	− 16	− 66	18	5.04	1732
Extra-nuclear area	− 22	0	− 8	4.90	1167
Left superior temporal gyrus (BA22)	− 44	− 54	16	4.62	1282
Right primary somatosensory cortex (BA3)	46	− 22	40	4.49	96
Left medial dorsal thalamic nucleus	− 4	− 12	8	4.04	179
Left parahippocampal gyrus (BA36)	− 22	− 40	− 8	3.92	116
**(C) Increased activation during negative attentional bias (*fear* > *happy*)**					
Right putamen	30	0	2	4.33	89
Left insula	− 24	26	16	4.18	885
Left putamen	− 20	− 14	2	3.81	16
Right parahippocampal gyrus (BA36)	26	− 32	− 6	4.16	60
Right midbrain	14	− 10	− 2	3.94	32
Brainstem, pons	− 4	− 24	− 16	3.52	27
Sub-gyral region	− 42	− 2	− 20	3.70	10
Right precuneus	24	− 46	0	3.59	46
Right precentral gyrus (BA6)	38	− 18	50	3.61	143
Right middle frontal gyrus (BA46)	42	16	30	3.59	23

Significant activation clusters are shown for the main effects of congruency and emotion. No decreased activation during negative attentional bias (fear < happy) was observed. All reported clusters met a voxel-level threshold of uncorrected *p* < 0.001 together with the Monte Carlo (3dClustSim)-derived minimum cluster extent of *k* ≥ 10 voxels, which corresponds to a cluster-level family-wise-error-corrected *p* < 0.05 for the present data

**Fig. 2:**
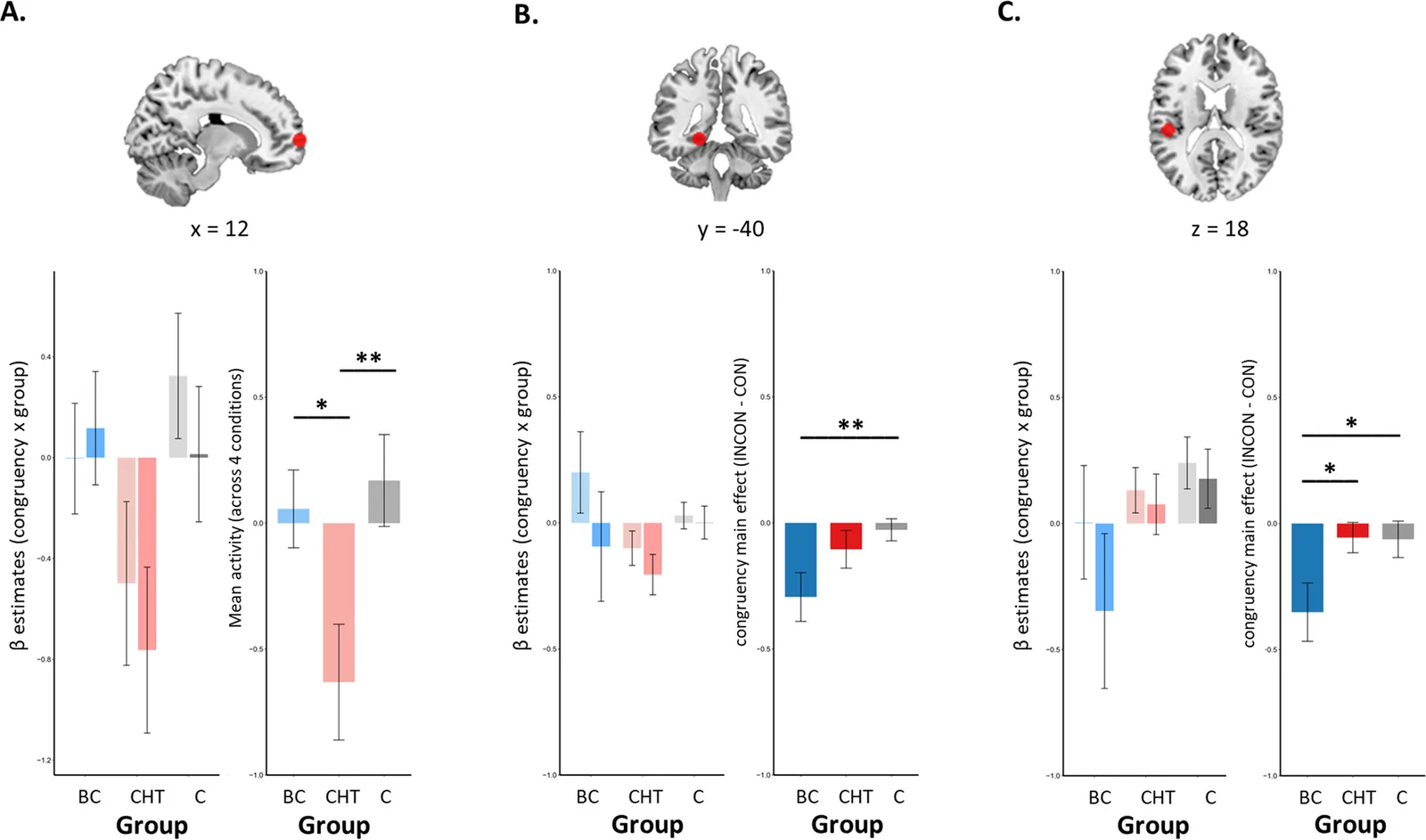
Neural activation in regions of interest (ROIs). **A** Dorsomedial prefrontal cortex (DMPFC) region of interest, a 10-mm-radius sphere centered on the a priori meta-analytic coordinate MNI (6, 62, 14) as specified in the “Methods” section, showing a main effect of group (*F*(2,67) = 4.160, *p* = 0.020; FDR-corrected *q* = 0.045). The section displayed is centered at MNI (12, 66, 4), the local activation maximum used for visualization; all reported statistics derive from the a priori sphere. **B** Left parahippocampal region of interest (10-mm sphere centered on the a priori coordinate MNI − 12, − 44, − 2; displayed section at MNI − 22, − 40, − 8, which corresponds to the independent whole-brain peak listed in Table 2) and (**c**) left insula region of interest (10-mm sphere centered on the a priori coordinate MNI − 34, − 30, 22; displayed section at MNI − 48, − 28, 18), showing group × congruency effects (parahippocampus: *F*(2,67) = 3.047, *p* = 0.054, *q* = 0.054; insula: *F*(2,67) = 3.283, *p* = 0.044, *q* = 0.054). Neither interaction survives FDR correction across the ROI effects, and both are presented descriptively. Left panels depict mean activation under different congruency conditions for each group: left bars represent congruent trials; right bars represent incongruent trials. Only within-group post hoc comparisons are denoted. Right panels show group-wise mean activation or interference effect (incongruent–congruent trials). Group shown from left to right: BC, CHT, C. Data are mean ± S.E.M. Asterisks indicate significant differences between groups: *p* < 0.05 (*), *p* < 0.01 (**)

ROI analyses (age as a covariate) revealed a significant group effect in DMPFC (*F*(2,67) = 4.16, *p* = 0.020, ηp2 = 0.11; FDR-corrected *q* = 0.045): CHT showed reduced activation (–0.857 ± 0.202) compared with both BC (–0.097 ± 0.291) and controls (–0.12 ± 0.192) (*p* < 0.05; Fig. 2A).

In the parahippocampal cortex, there was a significant congruency effect (*F*(1,67) = 11.73, *p* = 0.001, ηp2 = 0.15; *q* = 0.004) and a group × congruency interaction that did not reach significance (*F*(2,67) = 3.05, *p* = 0.054; *q* = 0.054) and is therefore described descriptively rather than treated as an established effect. The congruency effect was largest in BC (INCON vs. CON: –0.093 vs. 0.201, *p* < 0.05), smaller in CHT (–0.204 vs. –0.100, n.s.), and minimal in controls (0.001 vs. 0.029, n.s.) (Fig. 2B).

The insula showed a congruency main effect (*F*(1,67) = 10.97, *p* = 0.001, ηp2 = 0.14; *q* = 0.004) and a group × congruency interaction (*F*(2,67) = 3.28, *p* = 0.044, ηp2 = 0.089; *q* = 0.054, not surviving FDR correction across ROI effects): congruency effect greatest in BC (–0.347 vs. 0.004, *p* < 0.05), attenuated in CHT and controls (both n.s.) (Fig. 2C).

**Fig. 3:**
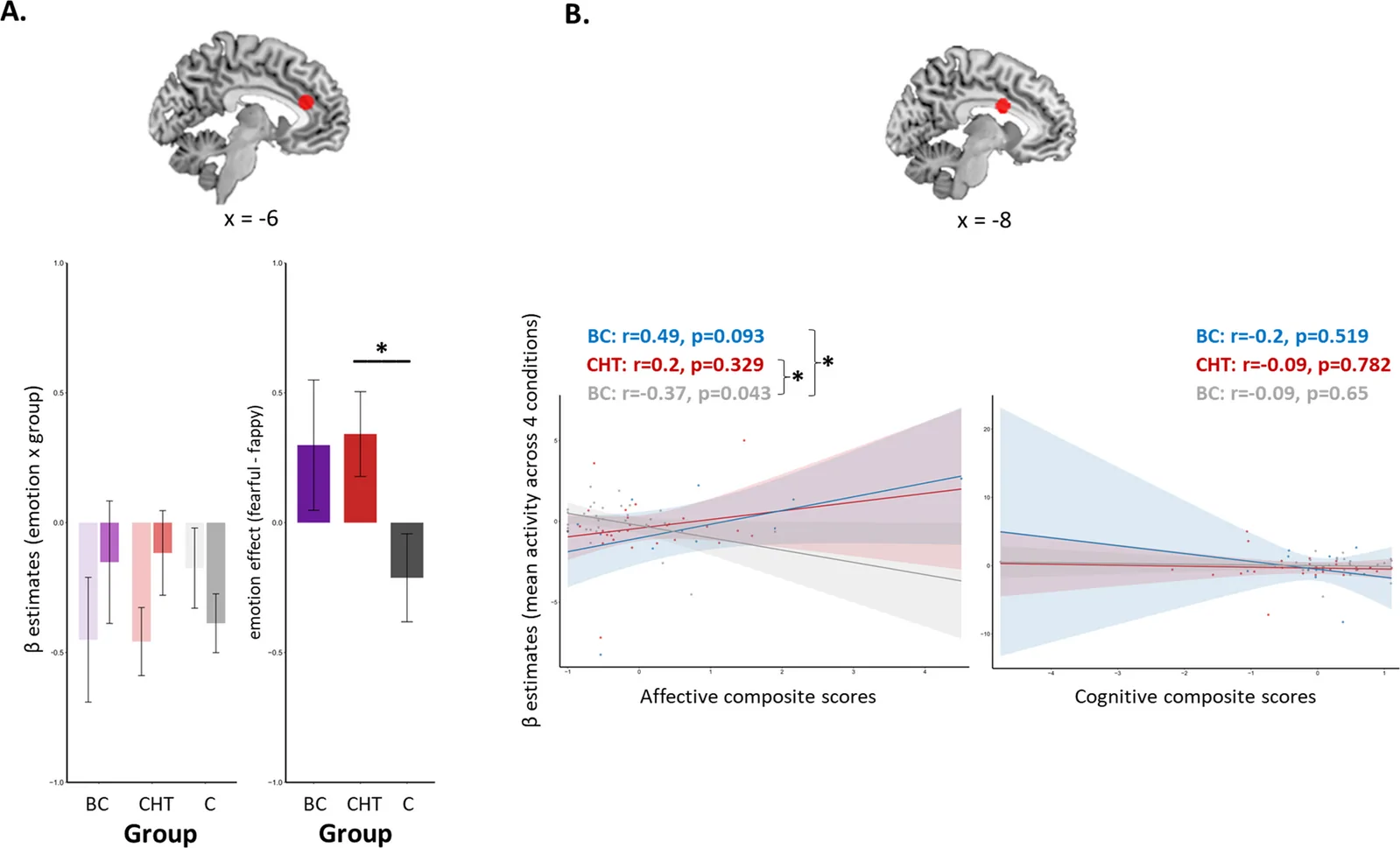
Dorsal anterior cingulate cortex activation and neural correlations. **A** Group × emotion effect in the left dorsal anterior cingulate cortex (dACC) region of interest, a 10-mm sphere centered on the a priori coordinate MNI (−6, 14, 26) as specified in the “Methods” section (*F*(2, 67) = 3.124, *p* =.050; *q* =.054, not significant after FDR correction and shown for descriptive purposes). The section displayed is centered at MNI (−6, 34, 20). Left panel: bars represent dACC activation during happy (left bar) and fearful (right bar) trials. Right panel: activation during negative attentional bias, calculated as fearful minus happy trials. **B** Correlation between activation in the same a priori left dACC sphere (center MNI −6, 14, 26; displayed section at MNI −8, 4, 28) and affective or cognitive index scores. A significant negative correlation was found between dACC activation and affective index in the control group (*r* =  − 0.37, *p* =.043). Fisher’s *r*-to-*z* transformation revealed significant group differences in affective correlations between the BC and control groups (*z* = 2.49, *p* =.013*) and between the CHT and control groups (*z* = 2.10, *p* =.036*). No significant group differences were observed for correlations with the cognitive index

The dACC showed a congruency main effect (*F*(1,67) = 5.19, *p* = 0.026, ηp2 = 0.07; *q* = 0.045) and a group × emotion interaction at the significance boundary (*F*(2,67) = 3.12, *p* = 0.050; *q* = 0.054, not surviving correction), reported here descriptively. The fearful-versus-happy difference was greatest in CHT (−0.116 vs. −0.458, *p* < 0.05), smaller in BC and reversed in controls (Fig. 3A). A negative correlation between affective index and mean dACC activity was observed only in controls (*r* =  − 0.371, *p* = 0.043), not in BC (*r* = 0.49, *p* = 0.093) or CHT (*r* = 0.20, *p* = 0.329). Fisher’s *r*-to-*z* tests indicated different association patterns (Control vs. BC: *Z* = 2.49, *p* = 0.013; Control vs. CHT: *Z* = 2.10, *p* = 0.036) (Fig. 3B). Because these comparisons rest on small subgroups, the correlation comparisons are regarded as exploratory throughout. No dACC-cognitive-index associations were significant.

## Discussion

Breast cancer survivorship care increasingly demands that clinicians identify and support survivors whose emotional needs may not be visible through conventional self-report screening. Yet a persistent puzzle in the supportive care literature—why some survivors report elevated distress while others appear remarkably resilient, particularly after chemotherapy—has limited the precision of psychosocial triage. To our knowledge, the present study provides the first task-based fMRI evidence obtained with an affective paradigm specifically designed to probe emotional attention and conflict processing in breast cancer survivors, identifying distinct neurobehavioral phenotypes that help reconcile these contradictory trajectories and carry direct implications for how supportive care is delivered.

### Summary of key findings

At the clinical assessment level, the BC group reported significantly higher affective symptoms than both CHT and controls, with no group differences on the cognitive index. This alone would lead a supportive care team relying solely on self-report to prioritize non-chemotherapy survivors for psychosocial intervention and deprioritize chemotherapy-exposed survivors—a triage decision our neural findings call into question. On the Stroop task, all participants were slower and less accurate for incongruent versus congruent trials and for fearful versus happy faces [12, 36]. Critically, CHT showed significantly lower accuracy than controls, particularly for fearful faces, despite reporting lower affective symptoms than BC—an early signal that low self-reported distress in CHT may coexist with compromised emotional processing.

Neuroimaging converged on this interpretation. CHT-specific DMPFC hypoactivation and markedly attenuated incongruency-related interference in parahippocampal cortex and insula distinguished CHT from BC, which retained robust incongruency effects. The normative inverse coupling between dACC activation and affective symptom severity was present only in controls, lost in both survivor groups. The DMPFC supports higher-order cognitive control and emotion regulation [12, 30]; reduced DMPFC engagement in CHT aligns with prior evidence identifying prefrontal cortex as a key target of chemotherapy-related neurotoxicity [22, 28].

### Emotional blunting as a mechanism with supportive care consequences

The most clinically consequential finding is that CHT’s lower self-reported symptoms were paralleled by—not despite—reduced neural engagement in conflict-monitoring and emotional attention regions. This pattern is consistent with, but does not establish, our emotional attention disruption hypothesis: chemotherapy-related alterations within salience network nodes (insula, parahippocampal cortex, DMPFC, ACC) may reduce the capacity to detect, prioritize, and respond to emotionally salient stimuli. Behaviorally, this would manifest as reduced interference from negative distractors, subjectively, as lower distress on standard screening. One interpretation of this configuration, taken together with the loss of the normative ACC-distress coupling, is emotional blunting, that is, a dampening of affective reactivity across neural, behavioral, and subjective levels. We emphasize, however, that emotional blunting was not measured directly here. Alternative accounts—including reduced task engagement, altered semantic-visual conflict resolution, and chemotherapy-related cognitive inefficiency—cannot be excluded, and low self-reported distress in individual survivors may still reflect genuine psychological adaptation.

Chemotherapy may therefore produce apparent emotional stability indirectly, by lowering emotional attention and thereby mimicking resilience on self-report [17, 18, 25]. This mechanism aligns with ERP findings of delayed N1 and P2 components in breast cancer survivors, indicating slowed early-stage emotional processing [14]. For oncology supportive care providers, this raises a clinically important caution: low distress scores in chemotherapy-exposed survivors may not always index authentic well-being and warrant attentive follow-up rather than automatic reassurance that psychosocial support is unnecessary. It should also be stressed that the present design cannot test the pathway implied by this account, in which chemotherapy exposure alters neural engagement during emotional conflict and thereby lowers self-reported distress. A formal mediation or pathway analysis would be required, and we deliberately refrained from conducting one because the modest and unequal group sizes, particularly the non-chemotherapy subgroup (*n* = 13), would render indirect-effect estimates unstable. The proposed pathway therefore remains inferential and requires confirmation in larger longitudinal samples; accordingly, we avoid causal and mediational language throughout.

### Neural substrates linking cognitive and affective dimensions

DMPFC hypoactivation in CHT is noteworthy given this region’s integration of cognitive control with affective salience. Reduced DMPFC recruitment during emotional conflict may impair top-down modulation of emotional interference, contributing both to poorer fearful-face accuracy and, clinically, to everyday difficulties regulating emotion under stress [22, 28].

The parahippocampal gyrus supports encoding of emotionally salient memories and contextual associations [1] and has been linked to affective disturbances including negative rumination. The insula, central to interoceptive awareness and emotion regulation [27, 32], shows abnormal activation in mood and anxiety disorders and is implicated in recognition of negative emotions [4]. The attenuated congruency effect in both regions among CHT may reflect the neural footprint of emotional blunting.

The dACC findings add further complexity. In controls, greater dACC activation correlated with lower affective symptoms, consistent with its role in conflict monitoring and regulatory control [11, 19]. This brain–behavior link was absent in both survivor groups, suggesting a decoupling of dACC regulatory function from affective outcomes after diagnosis or treatment. In CHT, the largest fearful-versus-happy dACC difference coincided with reduced fearful-trial accuracy. Given the dACC’s role in error-related negativity (ERN) processing, one speculative possibility is amplified ERN signalling, that is, an inefficient overinvestment of cognitive resources rather than effective regulation. No electrophysiological data were collected in the present study, so this should be read strictly as a hypothesis to be tested in future combined fMRI-EEG work, and not as a conclusion supported by the current data. For supportive care, this decoupling signals that even when survivors appear behaviorally comparable to controls, the neural economy supporting their emotion regulation may be fundamentally altered.

### Clinical implications for personalized survivorship care

These findings position divergent survivor outcomes along a single emotional attention continuum: hyper-reactivity (as in BC) increases vulnerability to anxiety, depression, and stress, whereas hypo-reactivity (as in CHT) lowers subjective distress at the cost of reduced neural and behavioral sensitivity to affective information. Both deviate from the balanced reactivity seen in healthy controls. This continuum offers supportive care teams a mechanistic framework for interpreting discrepant affective outcomes and tailoring intervention accordingly.

Three implications follow, which we frame as hypotheses for evaluation rather than as evidence-based clinical recommendations, since no intervention data were collected here. First, re-interpreting distress screening: low self-report distress in CHT should not be equated with intact emotional health. Teams may benefit from supplementing standard instruments (BAI, BDI-II, Distress Thermometer) with behavioral probes of emotional attention; at minimum, documenting chemotherapy history alongside distress scores can avoid under-triaging CHT survivors whose low scores mask neural compromise. Second, treatment history-informed intervention targeting: for CHT, approaches that re-engage emotional attention—emotion-focused therapy, exposure-based approaches, affect-labeling training—may restore adaptive salience detection while protecting social functioning and empathy, which blunting can quietly erode. For non-chemotherapy survivors showing hyper-reactive salience processing, mindfulness-based stress reduction, cognitive-behavioral therapy, and attention-bias modification may more effectively dampen reactivity. A single standardized protocol for all survivors is thus likely suboptimal. Third, integrating neurocognitive profiling: while task-based fMRI is not scalable for routine clinics, behavioral proxies of emotional attention (e.g., emotional Stroop performance) may be feasible adjuncts to distress screening, helping stratify survivors for mechanism-matched support. We stress that whether either strategy actually improves outcomes in the corresponding phenotype is untested; these proposals require randomized supportive care trials before clinical adoption, and the affective re-engagement strategies suggested for chemotherapy-exposed survivors in particular should be regarded as a hypothesis generated by, not demonstrated by, the present data.

### Limitations

Several limitations qualify these findings. The cross-sectional design precludes causal inference; longitudinal studies following survivors from pre-treatment into long-term survivorship are needed. The modest sample size, though typical for neuroimaging in cancer research, limited power to detect subtle effects. With 13 participants, effect-size estimates are unstable and their confidence intervals wide, so these effects should be treated as preliminary and requiring replication in an independent, adequately powered sample; we therefore report FDR-corrected q values for all ROI effects and describe the interactions that do not survive correction as descriptive rather than established. Survivors were also recruited from a single hospital clinic, which introduces potential selection bias: clinic-attending survivors may differ systematically from community-dwelling survivors in help-seeking behavior, symptom burden, and follow-up adherence, limiting generalizability.

Second, emotional blunting was inferred rather than measured: no trial-wise valence or arousal ratings and no indices of alexithymia, anhedonia, interoceptive awareness, or emotional avoidance were obtained. Blunting is therefore offered as a hypothesis compatible with the observed pattern, and future work should add explicit affective ratings and blunting-sensitive instruments.

Third, the face-word Stroop task assumes that standardized facial configurations map onto discrete emotion categories, an assumption that is contested: forced-choice formats and posed photographs inflate apparent universality [29], facial movements do not uniquely specify internal states [3, 8], and emotion perception from faces is not culturally universal [15]. Reduced interference on fearful-face trials may therefore reflect attenuated processing of socially learned emotion categories, lower task engagement, or chemotherapy-related cognitive inefficiency rather than blunting per se; our findings pertain to emotional-attentional processing of normatively categorized stimuli.

Fourth, the pathway from chemotherapy exposure through altered neural engagement to lower distress was not tested formally, so mediation is not demonstrated. Fifth, accuracy was near ceiling in all groups, rendering the behavioral findings conservative. Sixth, Fisher’s *r*-to-*z* comparisons are sensitive to influential observations in small samples, so they are treated as exploratory. Seventh, the low distress in CHT may partly reflect regression to the mean, which only pretreatment baselines can exclude. Finally, other paradigms and modulators such as fatigue, sleep, hormonal change, and inflammation warrant study.

## Supplementary Information

Below is the link to the electronic supplementary material.

ESM 1 (DOCX 30.9 KB)

## Data Availability

All data generated or analyzed during this study are included in this article. Further enquiries can be directed to the corresponding author.
