# Supplementary material for: Distinct affective neural phenotypes in breast cancer survivors: implications for personalized supportive care after chemotherapy
Source: Support Care Cancer. 2026 Oct 2;34(10):1048. doi: 10.1007/s00520-026-11271-z (PMC13633325; doi:10.1007/s00520-026-11271-z)
Supplement: Supplementary file 1 [file 520_2026_11271_MOESM1_ESM.docx]

**Supplementary Materials**

*Distinct affective neural phenotypes in breast cancer survivors: implications for personalized supportive care after chemotherapy*

**S1. Supplementary Methods**

**S1.1 MRI image acquisition**

Both structural and functional MRI data were acquired in the Brain Connectivity Laboratory of National Yang Ming Chiao Tung University using a 3T Siemens Magnetom Tim Trio scanner (Erlangen, Germany) equipped with a 32-channel head array coil. Functional images were collected along the AC–PC plane, and blood-oxygen-level-dependent (BOLD) T2*-weighted MR signals were measured by a gradient echo-planar imaging (EPI) sequence with the following parameters: TR = 2200 ms, TE = 30 ms, FOV = 220 × 220 mm², flip angle = 90°, matrix size = 64 × 64, 36 transverse slices, voxel size = 3.4 × 3.4 × 3.0 mm³, no gap. High-resolution structural images were acquired using a 3D magnetization-prepared rapid gradient-echo sequence (MPRAGE): TR = 2530 ms, TE = 3.5 ms, FOV = 256 × 256 mm², flip angle = 7°, TI = 1100 ms, matrix size = 256 × 256, 192 sagittal slices, voxel size = 1.0 × 1.0 × 1.0 mm³, no gap.

**S1.2 fMRI preprocessing**

fMRI data were preprocessed using MATLAB R2023a (MathWorks Inc., Natick, MA) with the Statistical Parametric Mapping toolbox (SPM12; Wellcome Centre for Human Neuroimaging, UCL). EPI images were first reoriented to each participant's T1 image, then normalized into Montreal Neurological Institute (MNI) stereotaxic space, and spatially smoothed with an 8-mm full-width-at-half-maximum (FWHM) Gaussian kernel. A high-pass frequency filter (128-s cutoff) was applied to the time series. Temporal autocorrelations were estimated using a first-order autoregressive model (AR[1]).

**S1.3 First-level GLM specification**

During first-level analysis of each participant, a voxel-wise generalized linear model was constructed for "on" block BOLD signal changes. Task events were modeled using boxcar regressors convolved with the canonical hemodynamic response function (HRF), comprising four task conditions: congruent happy, congruent fearful, incongruent happy, and incongruent fearful. Six movement parameters were included as nuisance regressors of no interest. In addition to the six realignment parameters, framewise displacement was computed from the realignment estimates and screened before second-level analysis; data were required to show mean framewise displacement < 0.5 mm and maximum translation/rotation < 3 mm/3°. No participant exceeded these criteria, so no participant was excluded on motion grounds and volume censoring (scrubbing) was not applied.

**S1.4 Second-level analyses and statistical thresholding**

Contrast images were entered into second-level random-effects analyses following the same factorial structure as the behavioral model, with group (CHT, BC, Control) as a between-subjects factor and congruency (congruent/incongruent) and emotion (happy/fearful) as within-subject factors. A mixed-model ANOVA examined main effects and interactions. Group-level covariate analyses assessed how brain activation patterns related to cognitive and affective composite indices. Fisher's r-to-z transformation and subsequent Z-tests were used to compare correlation coefficients between groups. Because the three groups differed significantly in age, age was entered as a nuisance covariate in every second-level model, for both the whole-brain and the ROI analyses. Main effects and interactions were each thresholded and corrected within their own contrast rather than jointly across the family of contrasts, and the results are interpreted accordingly. Because the survivor subgroups were small, all correlation comparisons are treated as exploratory rather than confirmatory.

Statistical thresholds for whole-brain analyses were set at voxel-level p < 0.001 (uncorrected), with a cluster-extent threshold determined via Monte Carlo simulation using 3dClustSim (https://afni.nimh.nih.gov/pub/dist/doc/program_help/3dClustSim.html), yielding a family-wise error (FWE)–corrected threshold of p < 0.05 that accounted for spatial autocorrelation across neighboring voxels. With the smoothness of the present data (8-mm FWHM Gaussian smoothing kernel), the voxel size, first-nearest-neighbour clustering, and the whole-brain search volume, 3dClustSim returned a minimum cluster extent of k = 10 voxels as the cluster-extent threshold required for a cluster-level FWE-corrected α < 0.05 at a voxel-level threshold of p < 0.001. All clusters reported in Table 2 of the main text therefore satisfy this corrected threshold, and clusters close to the extent threshold are interpreted with corresponding caution. Anatomical labels for whole-brain clusters were assigned with the Talairach Daemon following MNI-to-Talairach transformation; labels such as "Extra-Nuclear Area" and "Sub-Gyral Region" indicate white-matter or subcortical locations for which the atlas returns no gyral label.

**S1.5 Region-of-interest (ROI) definition**

Based on prior meta-analytic findings (Feng et al., 2018; Groenewold et al., 2013; Song et al., 2017), ROIs were defined as spherical regions within 10 mm of reported peak coordinates. ROIs included the dorsomedial prefrontal cortex (DMPFC; MNI: x = 6, y = 62, z = 14), dorsal anterior cingulate cortex (dACC; MNI: x = –6, y = 14, z = 26), parahippocampal cortex (MNI: x = –12, y = –44, z = –2), and left insula (MNI: x = –34, y = –30, z = 22). ROI-based analyses were performed using the MarsBaR toolbox within the SPM12 environment. A 10-mm sphere radius was adopted for three reasons: it approximates the spatial uncertainty associated with meta-analytic peak coordinates, it is commensurate with the 8-mm FWHM smoothing kernel applied to the functional data, and smaller spheres would sample fewer effectively independent voxels than the effective resolution of the smoothed data supports. Despite this radius, the four spheres did not overlap: the pairwise centre-to-centre separations were DMPFC-dACC 50.9 mm, DMPFC-parahippocampal 108.7 mm, DMPFC-insula 100.6 mm, dACC-parahippocampal 64.7 mm, dACC-insula 52.3 mm, and parahippocampal-insula 35.4 mm, so the smallest separation (35.4 mm) exceeded the 20 mm required for two 10-mm spheres to be disjoint. All ROI coordinates quoted in the main text, the figure legends, and these Supplementary Materials refer to these a priori sphere centres; whole-brain peaks reported in Table 2 were obtained independently and may therefore differ from the sphere centres. Age was included as a covariate in the ROI second-level models, exactly as in the whole-brain analyses. Because four ROIs were interrogated, the seven reported ROI omnibus effects were additionally evaluated with Benjamini-Hochberg false-discovery-rate (FDR) correction; both uncorrected p values and FDR-corrected q values are reported in the main text. Under this correction the parahippocampal congruency effect (q = 0.004), the insular congruency effect (q = 0.004), the DMPFC group effect (q = 0.045), and the dACC congruency effect (q = 0.045) remained significant, whereas the three group × condition interactions (q = 0.054) did not and are accordingly described descriptively rather than as established effects.

**S1.6 Behavioral data analysis — additional details**

Behavioral analyses were performed using SPSS version 24.0 (IBM Corp., Armonk, NY, USA). Trials classified as errors or those exhibiting RTs outside the 300–4000 ms range were excluded from analysis (<0.5% of the total dataset). A mixed-design ANOVA was conducted with group (CHT, BC, Controls) as a between-subjects factor and congruency (congruent/incongruent) and emotion-of-face (happy/fearful) as within-subject factors. Where applicable, one-way ANOVAs and independent-samples t-tests were used for post hoc comparisons. Pearson's correlation analyses examined associations between neuropsychological measures and behavioral indices. Statistical significance was defined as p < 0.05 for all tests. Because group sizes were unequal, the homogeneity-of-variance assumption was examined with Levene’s test, sphericity was assessed with Mauchly’s test, and the Greenhouse-Geisser correction was applied where sphericity was violated. For the BAI, whose variance differed most markedly across groups (SD 13.60 in BC vs. 4.52 in controls), Levene’s test confirmed heterogeneity of error variance, F(2, 67) = 10.82, p < 0.001, and the distribution was strongly right-skewed across the whole sample (n = 70, mean 7.56, SD 8.55, median 5.00, interquartile range 2.00–10.00, observed range 0–47). Two assumption-free sensitivity analyses were therefore performed, and both reproduced the parametric group effect. Welch’s robust test of equality of means, which does not assume equal variances, yielded F(2, 26.12) = 6.93, p = 0.004. The rank-based Kruskal-Wallis test yielded χ²(2) = 12.04, p = 0.002, with mean ranks of 44.00 in BC, 42.17 in CHT, and 25.82 in controls, preserving the group ordering obtained parametrically (BC ≥ CHT > controls). The elevated anxiety of both survivor groups relative to controls is therefore robust to the observed variance heterogeneity and non-normality, and it is not an artefact of the parametric model.

**S1.7 Stimulus set and randomization**

The stimulus set comprised 20 photographs drawn from the Pictures of Facial Affect: 10 identities displaying a prototypically happy expression and 10 identities displaying a prototypically fearful expression, selected on the basis of the normative recognition rates reported for the validated set and previously used with this paradigm (Chen et al., 2023). The identical stimulus set was presented to every participant, so that stimulus sampling was held constant across groups. Trial order within each block, and the pairing of happy or fearful emotion words with individual face photographs, were pseudorandomized for each participant under the constraint that each face appeared equally often in congruent and incongruent trials across the task; response-hand mapping was counterbalanced across participants. Throughout the manuscript, faces described as "happy" or "fearful" denote standardized stimuli normatively categorized as such rather than verified internal emotional states of the depicted individuals.

**S2. Supplementary Table**

**Supplementary Table S1. Post hoc pairwise comparisons (p values) for the affective index, BDI-II, and BAI across groups.**

*Index score annotations: a–c, BC > Control; d, CHT > Control; e, BC > CHT. Comparisons are derived from the one-way ANCOVA models with age as a covariate, computed on the same raw scores tabulated in Table 1 of the main text; the direction of every comparison is therefore consistent with Table 1, Figure 1A, and the Results text. Because the omnibus test for the BDI-II did not reach significance (p = 0.084 in Table 1), the corresponding pairwise values are reported for completeness and should be read descriptively rather than as confirmatory tests.*

|  | **BC vs C** | **CHT vs C** | **BC vs CHT** |
| --- | --- | --- | --- |
| Affective Index | 0.005 ** *^a^* | 0.100 | 0.046 * *^e^* |
| BDI-II | 0.020 * *^b^* | 0.645 | 0.056 |
| BAI | 0.002 ** *^c^* | 0.010 ** *^d^* | 0.060 |
